# Neuromodulated Spike-Timing-Dependent Plasticity, and Theory of Three-Factor Learning Rules

**DOI:** 10.3389/fncir.2015.00085

**Published:** 2016-01-19

**Authors:** Nicolas Frémaux, Wulfram Gerstner

**Affiliations:** School of Computer Science and Brain Mind Institute, School of Life Sciences, École Polytechnique Fédérale de LausanneLausanne, Switzerland

**Keywords:** STDP, plasticity, neuromodulation, reward learning, novelty, spiking neuron networks, synaptic plasticity (LTP/LTD)

## Abstract

Classical Hebbian learning puts the emphasis on joint pre- and postsynaptic activity, but neglects the potential role of neuromodulators. Since neuromodulators convey information about novelty or reward, the influence of neuromodulators on synaptic plasticity is useful not just for action learning in classical conditioning, but also to decide “when” to create new memories in response to a flow of sensory stimuli. In this review, we focus on timing requirements for pre- and postsynaptic activity in conjunction with one or several phasic neuromodulatory signals. While the emphasis of the text is on conceptual models and mathematical theories, we also discuss some experimental evidence for neuromodulation of Spike-Timing-Dependent Plasticity. We highlight the importance of synaptic mechanisms in bridging the temporal gap between sensory stimulation and neuromodulatory signals, and develop a framework for a class of neo-Hebbian three-factor learning rules that depend on presynaptic activity, postsynaptic variables as well as the influence of neuromodulators.

## 1. Introduction

After exposure to a stream of repetitive sensory inputs, e.g., cars passing by on a highway, humans do not remember each input configuration (every single car), but most often only a few relevant ones, such as the most salient, novel, or surprising items, e.g., a car involved in an accident. Similarly, after a set of attempts to solve a motor task, e.g., a child trying to open a bottle, the child does not memorize all the attempts that failed, but only the one that was rewarding. Reward, novelty or surprise are correlated with neuromodulatory signals, such as dopamine, acetylcholine or noradrenaline (Schultz, 2002; Ranganath and Rainer, 2003; Yu and Dayan, 2005). Dopamine is critical for the reinforcement of actions, consistent with theories of behavioral learning (Waelti et al., 2001; Steinberg et al., 2013) while several other neuromodulators are implicated in the creation of new memories (Gu, 2002; Hasselmo, 2006; Moncada and Viola, 2007).

Formation of new memories as well as the learning of actions or skills are thought to be linked to changes in synaptic connections (Hebb, 1949; Martin et al., 2000). Traditional approaches to synaptic plasticity, influenced by Hebb's postulate (Hebb, 1949), have focused on the joint activation of pre- and postsynaptic neurons as a driver for synaptic changes (Bliss and Gardner-Medwin, 1973; Artola and Singer, 1993; Malenka and Nicoll, 1999). Spike-Timing-Dependent Plasticity (STDP; Gerstner et al., 1996; Markram et al., 1997; Bi and Poo, 1998; Sjöström et al., 2001, can be considered as a temporally precise form of Hebbian synaptic plasticity, induced by isolated spikes in pre- and postsynaptic neurons (for reviews see e.g., Abbott and Nelson, 2000; Bi and Poo, 2001; Caporale and Dan, 2008; Sjöström et al., 2008; Sjöström and Gerstner, 2010; Markram et al., 2011). In many, but not all preparations, repeated activation of a presynaptic neuron a few milliseconds before the postsynaptic one yields potentiation of the synapse, whereas reverse timing yields depression (Abbott and Nelson, 2000). In theoretical models, this form of plasticity generates numerous attractive functional features (Gerstner et al., 1996; Kempter et al., 1999; Song et al., 2000; Song and Abbott, 2001; Clopath et al., 2010).

However, the functionality of STDP, and more generally that of Hebbian learning rules, is limited to the class of unsupervised learning tasks (Hertz et al., 1991). The aim of unsupervised learning is to adapt a system to the statistical properties of the environment. While unsupervised learning is one of the driving forces of developmental plasticity, Hebbian learning, STDP, as well as other unsupervised learning rules neglect, by design, any information regarding “reward,” “success,” “punishment,” or “novelty.” The question then arises of how neuromodulatory signals interact with neural activity to influence synaptic plasticity, learning, and ultimately behavior (Gu, 2002; Hasselmo, 2006; Calabresi et al., 2007).

Recently, a number of experimental studies have mastered the technical difficulties of controlling pre- and postsynaptic spiking activity, together with neuromodulator concentration, in order to study their combined effect on synaptic plasticity (Seol et al., 2007; Pawlak and Kerr, 2008; Shen et al., 2008; Pawlak et al., 2010). Parallel theoretical studies have explored on a more fundamental level the universe of synaptic plasticity rules that *could* potentially implement learning in neural circuits so that a formal neural network memorizes novel stimuli or sequences, (Brea et al., 2011; Rezende et al., 2011; Brea et al., 2013; Rezende and Gerstner, 2014) or learns rewarding skills (Xie and Seung, 2004; Pfister et al., 2006; Baras and Meir, 2007; Farries and Fairhall, 2007; Florian, 2007; Izhikevich, 2007; Legenstein et al., 2008; Di Castro et al., 2009; Potjans et al., 2009; Urbanczik and Senn, 2009; Vasilaki et al., 2009; Frémaux et al., 2010, 2013).

While the broader field of neuromodulation, plasticity, and behavioral learning has been reviewed before (Martin et al., 2000; Gu, 2002; Reynolds and Wickens, 2002; Schultz, 2002, 2006; Hasselmo, 2006; Shohamy and Adcock, 2010; Lisman et al., 2011; Nadim and Bucher, 2014), this review mainly focuses on the case of STDP under the influence of neuromodulation and its relation to models of learning. We first point out the limitations of standard Hebbian learning and sketch the concept of synaptic plasticity under the influence of neuromodulation. We then review experimental studies that combine the paradigm of STDP with neuromodulation. Finally, we summarize models of the combined action of neuromodulators and STDP in a unified theoretical framework and identify open questions for future experiments.

## 2. Basic concepts: hebbian and modulated hebbian plasticity

Behavioral learning and memory is thought to be linked to long-lasting synaptic changes (Hebb, 1949; Barnes, 1979; Morris et al., 1986; Bliss and Collingridge, 1993; Martin et al., 2000) that can be experimentally induced by protocols for long-term potentiation (LTP) (Lømo, 1964; Bliss and Lømo, 1973) and long-term depression (LTD) (Lynch et al., 1977; Levy and Stewart, 1983), or STDP (Markram et al., 1997; Bi and Poo, 1998; Sjöström et al., 2001). Before turning to experimental data of neuromodulated STDP, we discuss in this section the basic concepts of Hebbian learning (Hebb, 1949) that have influenced our current-day thinking about synaptic plasticity (Malenka and Nicoll, 1999; Bliss et al., 2003; Lisman, 2003).

Hebbian plasticity (Hebb, 1949) describes LTP of synapses that is induced by the *joint activation* of pre- and postsynaptic neurons (Brown et al., 1991; Gerstner et al., 2014). In order to formalize the idea of Hebbian plasticity, we denote the spike train of a presynaptic neuron by the short-hand notation “pre.” Similarly, the state of a postsynaptic neuron, including its (past) spike train, voltage, potentially intracellular calcium or other important variables, is summarized by “post.” In a mathematical notation, the change of a weight *w* from the presynaptic to the postsynaptic neuron during Hebbian learning can be described by

(1) $$\begin{array}{l} \dot{w}=H\left(\text{pre},\text{post}\right) \end{array}$$

where $\dot{w}$ describes the rate of change of the weight *w* and *H* is some arbitrary function of the presynaptic spike train and the state of the postsynaptic neuron.

Experimental support for Hebbian learning comes from observations that co-activation of pre- and postsynaptic neurons can induce LTP or LTD, depending on the relative firing frequency and timing of pre- and postsynaptic neurons (Levy and Stewart, 1983; Malenka and Nicoll, 1999; Abbott and Nelson, 2000; Bi and Poo, 2001; Markram et al., 2011) and voltage of the postsynaptic neuron (Artola and Singer, 1993; Sjöström et al., 2001; Sjöström and Gerstner, 2010). In other words, the activities of pre- and postsynaptic neurons are crucial factors for the induction of plasticity.

STDP is a typical example of Hebbian plasticity (Bi and Poo, 2001; Morrison et al., 2008). In the simplest model of STDP, the state of the postsynaptic neuron is characterized by its recent firing times. Equation (1) then indicates that changes of the synaptic weight depend on coincidences between the spikes of the pre- and postsynaptic neuron (Kempter et al., 1999; Song et al., 2000; Gerstner and Kistler, 2002; Morrison et al., 2008). Therefore, STDP, as well as other Hebbian learning rules, is sensitive to statistical correlations between neurons (Kempter et al., 1999; Gerstner et al., 2014). Using a standard classification from machine learning theory, we can state that Hebbian learning is “unsupervised” (Hertz et al., 1991; Gerstner et al., 2014), because it does not incorporate the notion of whether a synaptic change is useful or not.

From a theoretical perspective, unsupervised learning is a comparatively weak paradigm, since the class of learning problems that can be solved by unsupervised learning is limited. However, in view of the experimental results discussed in the next section, we may include in the mathematical picture one or several neuromodulators that would “gate” Hebbian plasticity such that up- or down-regulation of synapses happens at appropriate moments in time. If these neuromodulators convey information on novelty of (or surprise induced by) a stimulus or success of (or external reward in response to) an action, then the resulting learning rules are no longer “unsupervised,” but become more powerful. To illustrate the potential functions of plasticity under the influence of neuromodulators, we focus on two paradigms, reward-based learning and novelty-based learning.

### 2.1. Conceptual example: reward-based learning

**Figure 1:**
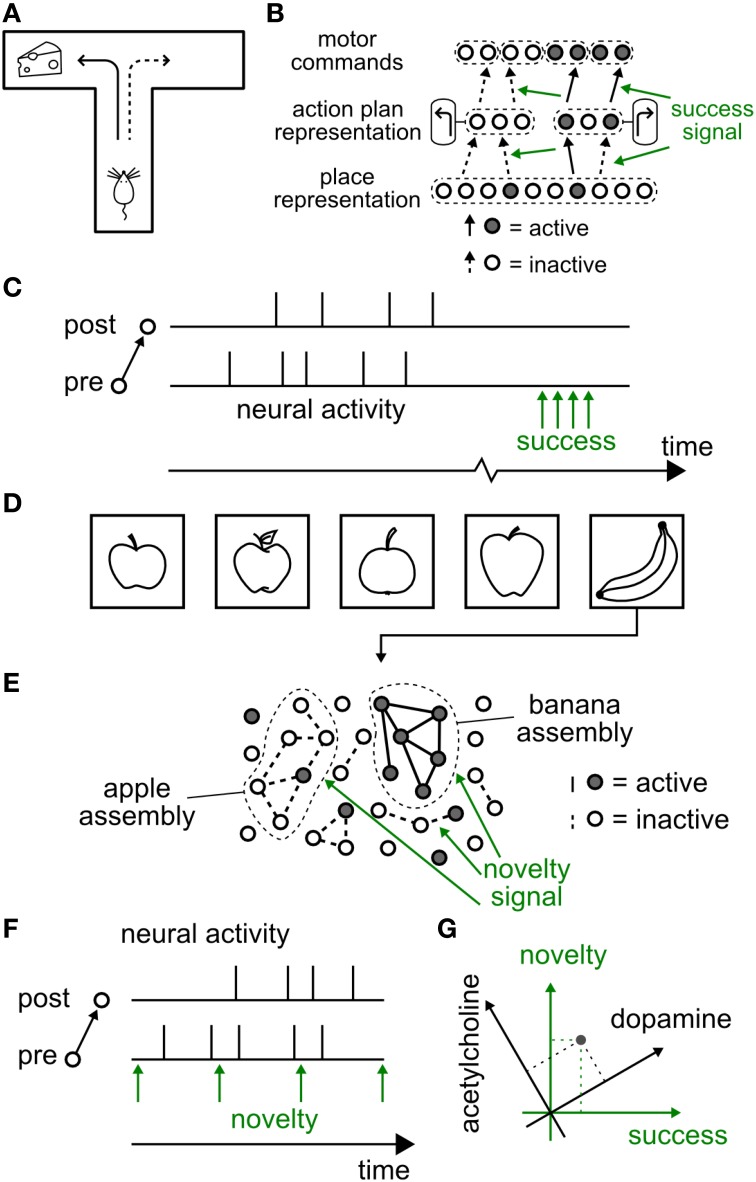
**Hypothetical functional role of neuromodulated synaptic plasticity**. **(A–C)** Reward-modulated learning **(A)** Schematic reward-based learning experiment. An animal learns to perform a desired sequence of actions (e.g., move straight, then turn left) in a T-maze through trial-and-error with rewards (cheese symbol represents location of reward). **(B)** The current position (“place”) of the animal in the environment is represented by an assembly of active cells in the hippocampus. These cells feed neurons (e.g., in the dorsal striatum) which code for high-level actions at the choice point, e.g., “turn left” or “turn right.” These neurons in turn project to motor cortex neurons, responsible for the detailed implementation of actions. A success signal, representing the outcome of the actions at the behavioral level (i.e., food or no food), modulates (green arrows) the induction of plasticity at those synapses that have been marked by coincident pre- and postsynaptic activity (solid black connections), but not of those synapses where either the pre- or the postsynaptic neuron was silent (dashed connections). Note that several intermediate layers are possible between each brain area. **(C)** Neuromodulatory timing. While action potentials of pre- and postsynaptic neurons occur on the time scale of milliseconds, the success signal, representing “reward minus expected reward,” occurs much later (broken time axis). **(D–F)** Novelty-modulated learning in a neural network. **(D)** Novelty is defined by the occurrence of a stimulus that does not match pre-existing experience. In this example, a neural network has been trained to recognize an apple. The first time it sees a banana recognition fails and a novelty signal is triggered. **(E)** Schematic of a neuromodulated neural network for novelty-based learning tasks. Neural assemblies represent known concepts. Here a “banana” stimulus is presented, failing to activate the “apple” neurons, but activating a group of other neurons which will, in the future, encode the “banana” concept. The novelty signal, concurrent with pre- and postsynaptic activation of the banana neurons ensures that synapses (solid lines) between neurons of the banana “assembly” are strengthened. The synapses of the “apple” assembly receive the same neuromodulatory signal, but do not change because pre- or postsynaptic neurons are not simultaneously active. **(F)** Neuromodulatory timing. Contrary to the reward-based case, the novelty signal can be synchronous with neural activity, or arise slightly earlier or later. **(G)** Schematic of relation between neuromodulators and functional roles. A specific neuromodulator (e.g., dopamine) could transmit a signal conveying a mixture of novelty and success (green axes). A novel event (gray dot) can be at the same time surprising and rewarding and cause the simultaneous emission of acetylcholine and dopamine, in different proportions (black axes).

In a schematic reward-based learning scenario (Arleo and Gerstner, 2000; Foster et al., 2000; Sheynikhovich et al., 2009; Frémaux et al., 2013), such as a T-maze (Figure 1A), the present position of the animal is represented by neuronal activity in the hippocampus (O'Keefe and Nadel, 1978; Moser et al., 2008). The animal's action at the choice point is represented by the activity of neurons in other brain areas, possibly including the dorsal striatum (Packard and McGaugh, 1992; Schmitzer-Torbert and Redish, 2004). Let us suppose that the animal decides to turn left (a decision represented in the conceptual model of Figure 1B by the activity of cell assemblies in the striatum) at the junction of a T-maze (a location represented in the model by cell assemblies in hippocampus). Thus, in that situation, several assemblies of neurons in different brain areas are co-activated. According to the principle of Hebbian learning, the co-activation of presynaptic neurons in hippocampus with postsynaptic neurons in the striatum drives learning. However, classical Hebbian learning cannot account for the fact that the reinforcement of the specific connection identified by the Hebbian co-activation principle must also depend on whether the action taken at the junction leads to a reward or not (Arleo and Gerstner, 2000; Foster et al., 2000; Xie and Seung, 2004; Pfister et al., 2006; Baras and Meir, 2007; Florian, 2007; Di Castro et al., 2009; Sheynikhovich et al., 2009; Urbanczik and Senn, 2009; Vasilaki et al., 2009; Frémaux et al., 2010, 2013).

The difference between a rewarded trial and an unrewarded one arises from information about the success of the action (e.g., a food reward) that is obtained by the animal in a given trial. Indeed, such a success signal is necessary for learning (Waelti et al., 2001; Steinberg et al., 2013). In neural network models of behavioral learning, such a success signal is exploited at the synaptic level to reinforce the correct sequence of actions (Figure 1B) by modulating Hebbian plasticity.

There is rich evidence for the neuromodulator dopamine to transmit a phasic success signal that is made available via ramified projections from dopaminergic neurons to several brain areas (Schultz, 1998, 2006). Here phasic means an activity peak that is precisely timed and relatively short compared to the total duration of an experimental trial. Note that the phasic success signal always arrives *after* the decision. The fact that a large delay can occur between the neural activity at the point of decision and the subsequent reward provides an additional difficulty (Figure 1C) which can be addressed either by Temporal Difference Learning (TD) (Schultz et al., 1997; Sutton, 1998; Sutton and Barto, 1998), or by eligibility traces (Baxter and Bartlett, 2001). Both types of model solutions will be discussed in Section 4.

### 2.2. Conceptual example: novelty-based learning

Another potential role for modulation of synaptic plasticity arises during learning of novel stimuli. A familiar visual stimulus which has already been seen several times does not require memorization if it is perceived again, whereas a stimulus that is novel and interesting should be stored (Carpenter and Grossberg, 1988).

We note that in standard Hebbian plasticity, every co-activation of pre- and postsynaptic neurons will potentially induce a further change in the connection. In this case, the memory capacity of model networks where stimuli or concepts are stored is rapidly reached such that old memories are overwritten by new ones (Fusi, 2002; Fusi and Abbott, 2007). To avoid constant overwriting of synaptic memories, it is desirable to limit the induction or expression of synaptic plasticity to the case of novel stimuli or concepts (Figure 1D). This could be achieved if plasticity of synapses requires pre- and postsynaptic activity *together* with a novelty signal (Figure 1E). The novelty signal could be encoded in the phasic activity of a neuromodulator that gates synaptic plasticity. In contrast to the reward-based learning scenario (where timing of a reward is delayed with respect to the act), the timing of a neuromodulatory signal for novelty could coincide with the period of increased neural activity (Figure 1F). Novelty (or surprise) is represented in the brain by multiple mechanisms and correlated with changes in the pupil size (Nasser et al., 2012) and the P300 component of EEG (Meyer et al., 1991; Kolossa et al., 2015). Novelty is also represented by the initial transient of the phasic dopamine signal (Schultz, 1998; Lisman et al., 2011) and by an increase in acetylcholine and noradrenaline (Ranganath and Rainer, 2003). While a direct interaction of acetylcholine with synaptic plasticity is one possibility (Gu, 2002; Hasselmo, 2006), acetylcholine can also affect learning of novel stimuli through several other mechanisms, such as enhancement of excitatory afferent input, suppression of excitatory feedback, modulation of theta rhythm, and increase of persistent spiking of individual cortical neurons (Hasselmo, 2006). Noradrenaline emission linked to arousal caused by novel stimuli could favor “the development of persistent facilitatory changes in all synapses that are currently in a state of excitation” as suggested by early conceptual theories (Kety, 1972); cited and evidence discussed in Sara (2009).

### 2.3. Conceptual role of neuromodulators in plasticity

Neuromodulators such as acetylcholine, noradrenaline, serotonin, dopamine (and potentially histamine) not only change the excitability of neurons (Kaczmarek and Levitan, 1987), but can also influence synaptic plasticity and memory formation (Rasmusson, 2000; Gu, 2002; Marder, 2012; Kang et al., 2014; Nadim and Bucher, 2014).

Dopamine signals have been linked to reward (Apicella et al., 1991; Schultz et al., 1997). Phasic responses of dopaminergic neurons in the macaque occur not only at the moments of rewards (Hollerman and Schultz, 1998) but also to stimuli that are predictive of reward (Schultz et al., 1997). This yielded an interpretation that dopaminergic neurons encode the “actual minus predicted reward” (Schultz, 2002).

Acetylcholine is necessary to induce plasticity in sensory cortices (Gu, 2002) and hippocampus (Drever et al., 2011) as shown in a variety of paradigms including sensory map remodeling in auditory cortex (Kilgard and Merzenich, 1998; Ma and Suga, 2005) or inhibitory avoidance training (Mitsushima et al., 2013). Similarly, noradrenaline and serotonin play a permissive and facilitatory role for the induction of plasticity (Seidenbacher et al., 1997; Gu, 2002; Tully and Bolshakov, 2010; Bergado et al., 2011). More recently, it has been shown that vagus nerve stimulation triggering release of a mix of neuromodulators gates plasticity (Engineer et al., 2011). Moreover, neuropeptides influence learning and plasticity (Hökfelt et al., 2000; Gøtzsche and Woldbye, 2015).

Even though dopamine is often associated with reward and acetylcholine with novelty, it is important to note that the mapping between neuromodulators and functional roles does not have to be one-to-one. Let us imagine that each functional signal (e.g., novelty) is carried by a linear or nonlinear combination of several neuromodulator concentrations (Figure 1G). For example, the time course of dopamine could contain information on a mixture of “reward compared to expected reward” and “novelty.” In parallel, a phasic acetylcholine signal could contain mixed information on surprise defined as “novelty compared to expected novelty” and “reward.” Some synapse types could use the combined action of both neuromodulators to drive novelty-based learning, while other synapse types in the same or other brain areas could use the combined action of both neuromodulators to drive reward-based learning. In addition, tonic and phasic components of the same neuromodulator could convey different functional signals (Lisman et al., 2011).

Moreover, other neuromodulators (Noradrenaline, Serotonin, …) and functional roles (stress, fear, and other emotions) could become part of the above picture. For example, noradrenaline-emitting neurons located in locus coeruleus and projecting axons to nearly all brain areas are reciprocally connected to dopaminergic neurons located in the VTA and are therefore likely to share some, but not all signaling features of dopamine-emitting neurons (Sara, 2009). Thus, the identification of a single neuromodulator with one isolated theoretical concept such as novelty or reward might be inappropriate.

**Figure 2:**
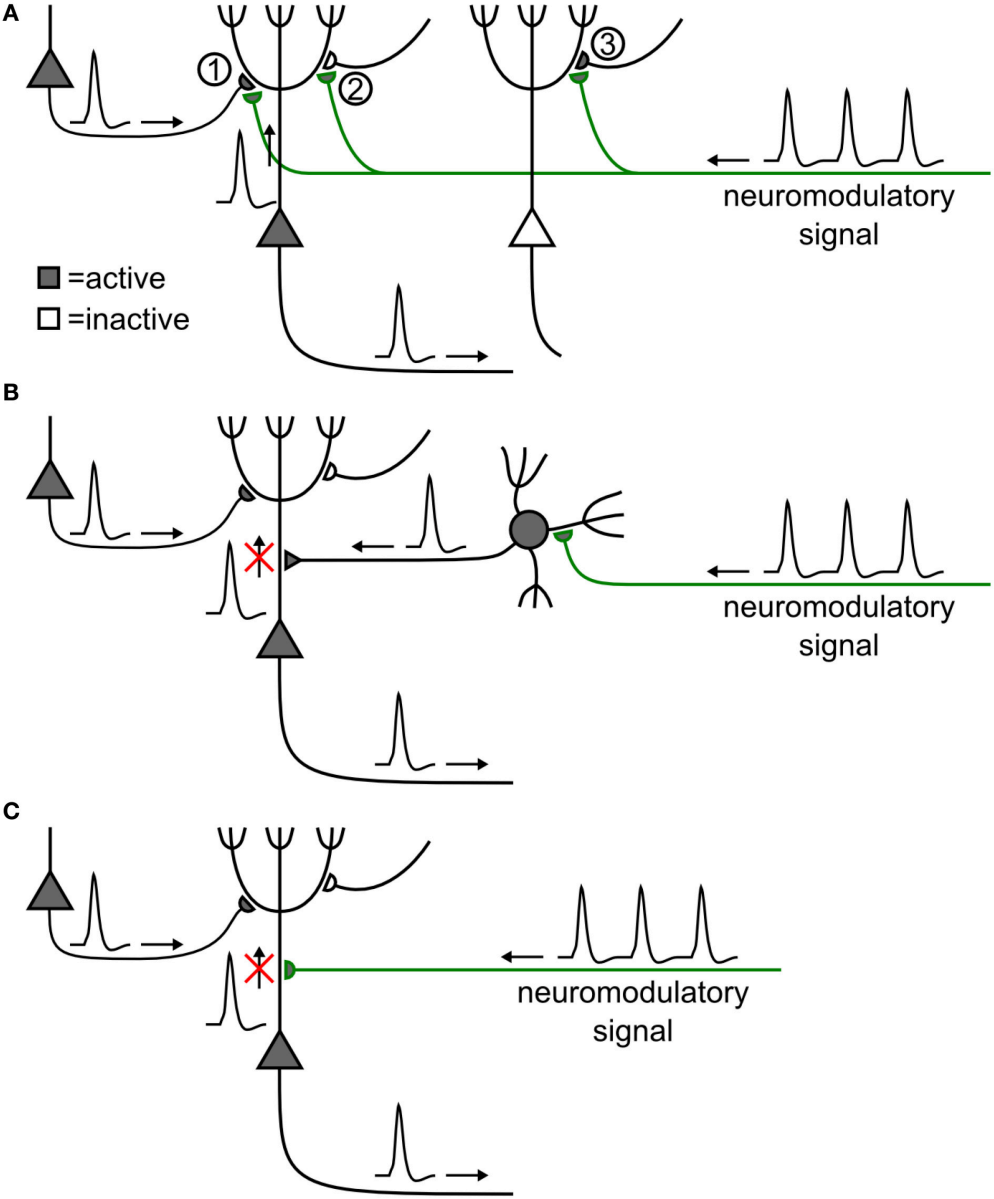
**Possible mechanistic scenarios of neuromodulation of synaptic plasticity**. **(A)** Direct synaptic effect (Goldman-Rakic et al., 1989). A synapse (1) between two excitatory pyramidal neurons (triangles) is strengthened by the coincident activity of its pre- and postsynaptic neurons, if neuromodulator is released. Plasticity is absent, weaker, or reversed, if the presynaptic (2) or the postsynaptic (3) neuron is silent (e.g., Schultz, 1998; Bailey et al., 2000). **(B)** Indirect effect of neuromodulation. Neuromodulator excites an inhibitory cell (filled circle), causing shunting inhibition on an excitatory neuron. This could prevent back-propagation of the action potential, thus blocking the induction of plasticity of a synapse, even though the presynaptic and postsynaptic neurons were active (e.g., Bissière et al., 2003). **(C)** Direct influence of neuromodulation on action potential back-propagation has a similar effect as in **(B)** (e.g., Sjöström et al., 2008).

STDP and phasic neuromodulatory signals could interact in different ways. The simplest picture is that of a direct interaction between synapses and neuromodulators (Figure 2A). An example could be the co-location of cortico-striatal synapses and dopaminergic terminals on the same spine of neurons in the striatum (Freund et al., 1984; Schultz, 1998, 2002). However, the involvement of GABAergic neurons raises the possibility that neuromodulation may affect synapses indirectly (Bissière et al., 2003), for example by allowing inhibition-mediated suppression of back-propagating action potentials (Figure 2B) known to influence the induction of STDP (Golding et al., 2002; Sjöström and Häusser, 2006). Neuromodulators influencing ion channels could affect action potential back-propagation (Figure 2C), or dendritic Ca^2+^ signaling, in turn modulating LTP and LTD induction (see e.g., Couey et al., 2007, for an example of acetylcholine modulation of STDP along these lines).

## 3. Experimental evidence for neuromodulation of STDP

STDP is induced by pairing protocols where pre- and postsynaptic spikes are induced in a controlled sequence (Markram et al., 1997; Bi and Poo, 1998; Sjöström et al., 2001). We review STDP experiments, where neuromodulation is manipulated during or after the pairing protocol.

### 3.1. Questions regarding modulated STDP

From the point of view of theoreticians, the concepts of Hebbian and modulated Hebbian learning that we have sketched in the previous section give rise to three questions that will be useful to frame our review of the experimental literature.

- What is the precise form of the *interaction* of a pairing protocol with a neuromodulatory signal? For example, does dopamine “gate” STDP induction (or classic LTP) in the sense that dopamine levels below a certain threshold block STDP altogether whereas super-threshold levels restore “normal” STDP? In this case we could describe the effect of dopamine as “permissive.” Or does the amount of STDP (or LTP) correlate with dopamine in the sense that a higher dopamine concentrations yields a stronger potentiation, i.e., a larger increase of the excitatory postsynaptic potential (EPSP)? In this case we could describe its effect as “multiplicative.”
- What is the relevant *neuromodulator variable* to consider? If phasic activity is defined as the momentary neuromodulator concentration close to a synapse minus a baseline, what happens if the concentration becomes smaller than the baseline concentration—does the induced change in synaptic strength switch its sign?
- What are the *timing* requirements of a phasic neuromodulatory signal? A standard STDP protocol of 60 pre-post pairings at 20Hz only takes a few seconds. Should the phasic neuromodulatory signal arise prior to the pre-/post-synaptic pairing? Is it necessary that the phasic neuromodulator signal is present during the pairing? Is it sufficient if it arrives only a few seconds after the pairing? How long should the neuromodulatory “pulse” be? Does precise timing matter at all, or is it sufficient for pairing and neuromodulator to co-occur within a larger time window, on the order of minutes to hours?

In the following, we review the published experimental results addressing the link between neuromodulators and STDP in light of these three questions (see also the summary in Table 1).

**Table 1:** **Selection of experimental results addressing the interaction of neuromodulation and STDP**.

		**Interaction type**	**Quantitative neuromodulation**	**Neuromodulator timing**	**Details**
Dopamine	Bissière et al., 2003	Inhibition-mediated gating	Baseline vs. bath application	5–10 min around stimulation	Lateral amygdala, mouse slice
	Pawlak and Kerr, 2008	Gating	Baseline vs. bath application	Always in bath	Corticostriatal, rat slice
	Shen et al., 2008	Gating	Baseline vs. bath application	Always in bath	Corticostriatal, mouse slice
	Zhang et al., 2009	Window shape alteration	Baseline vs. bath application	10 min around stimulation	Hippocampal culture, rat
	Xu and Yao, 2010	Inhibition-mediated gating	Baseline vs. bath application	5–10 min around stimulation	Prefrontal cortex, mouse slice
	Schulz et al., 2010	Unclear, gating of anti-Hebbian STDP?	Physiological via visual input and disinhibition of SC	100–250 ms after pairing	Corticostriatal, mouse Anesthesized
Non-dopamine	Lin et al., 2003	Window shape alteration	Baseline vs. bath application	Always in bath	Noradrenaline, hippocampus, rat
	Seol et al., 2007	Window shape alteration	Baseline vs. varying concentrations of two neuromodulators	10 min application, 10–60 min prior to stimulation	Acetylcholine and noradrenaline, visual cortex, rat
	Couey et al., 2007	Window shape alteration	Baseline vs. bath application	5–10 min around stimulation	Acetylcholine, prefrontal cortex, mouse

### 3.2. STDP protocols in conjunction with neuromodulators

In this subsection, we review experiments that study the interaction of neuromodulators with STDP. Little is known, however, about the molecular pathways or biophysical mechanisms that give rise to these interactions (see Pawlak et al., 2010, for a review).

The interaction of dopamine with STDP has been studied in several brain systems. In the amygdala, the link between dopamine and STDP is indirect via suppression of GABAergic inhibition (Bissière et al., 2003). However, suppressing GABAergic transmission altogether by pharmacological means prevents STDP induction, even in the presence of dopamine, suggesting a complex interplay between different factors.

In prefrontal cortex layer-5-pyramidal neurons, extracellular dopamine is necessary to induce LTP with pre-before-post pairings (Xu and Yao, 2010). Interestingly, STDP induced with repeated pre-before-post at 30 ms time difference requires activation of both D1 and D2 receptors, whereas for timings at 10 ms the activation of D2 receptors is sufficient, indicating multiple mechanisms of dopamine interactions: D2 receptors activation enables STDP by blocking inhibitory input whereas additional activation of postsynaptic D1 receptor extends the time window from 10 to 30 ms (Xu and Yao, 2010). For post-before-pre pairings, no information is available.

In corticostriatal synapses onto spiny projection neurons of the dorsal striatum, normal STDP is disabled by blocking D1/D5 (Pawlak and Kerr) or D2 receptors (Shen et al.). In both cases, a low baseline concentration (a few nM) of dopamine is likely to be present in the slice preparation, although the exact levels are not known. This “baseline” neuromodulator presence would then be responsible for STDP in the control condition. The results suggest an interaction of a “gating” type: dopamine permits STDP, while blocked dopamine receptors prevent STDP (Pawlak and Kerr, 2008). However, without additional dopamine concentration “data-points,” a multiplicative—or a more complicated—interaction pattern cannot be excluded. Moreover, the type of dopamine receptor at the postsynaptic site influences the plasticity pattern (Shen et al., 2008). The question of timing of a phasic neuromodulatory signal is left completely open by these studies, since they have been performed with constant extracellular bath concentration.

In striatal neurons of anesthetized mice *in vivo*, an intricate protocol to mimic natural phasic dopamine release and presynaptic activity leads to a small plasticity effect for very short timings (<5 ms) (Schulz et al., 2010).

**Figure 3:**
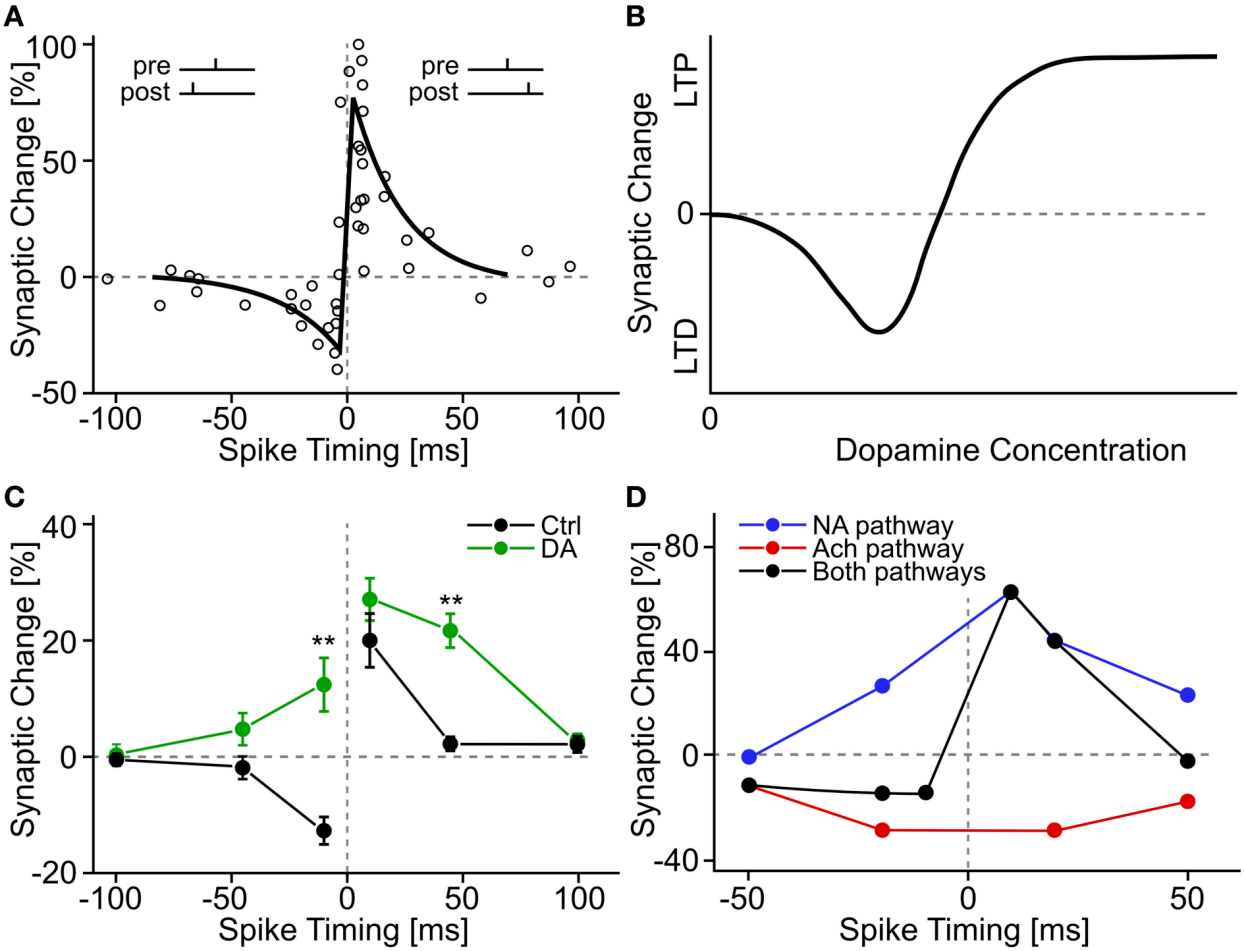
**Experimental evidence for STDP and neuromodulated STDP**. **(A)** A classic example of STDP measurement. Each circle shows a measurement of synaptic change as a function of the time difference between pre- and postsynaptic spikes. The black line shows a schematic fit of an “STDP window.” Data adapted from Bi and Poo (1998), hippocampal cell culture from rat. **(B)** Schematic summary effect of dopamine concentration on the induction of long-term plasticity in corticostriatal synapses. Adapted from Reynolds and Wickens (2002) who summarize data from many earlier experiments. **(C)** Effect of extracellular dopamine (DA) on the STDP window. Adapted from Zhang et al. (2009), hippocampal cell culture from rat. **(D)** Effect of activation of neuromodulatory pathways on the STDP window. The noradrenaline (NA) pathway is activated through β-adrenergic receptors, the acetylcholine (Ach) pathway through M1-muscarinic receptors. Adapted from Seol et al. (2007), rat visual cortex slices.

Cell cultures of rat hippocampal neurons devoid of dopaminergic cells exhibit a standard “STDP window” with pre-before-post pairings inducing LTP and post-before-pre pairings inducing LTD (Zhang et al., 2009). When dopamine is added to the extracellular bath, the authors find (i) stronger LTP, (ii) relaxation of the timing requirement for LTP (longer pre-post intervals would still yield LTP) and (iii) a sign-flip for post-before-pre timing from LTD to LTP (see Figure 3C). This study provides an example of a relation between dopamine and STDP that is more complex than the simple gating proposed in earlier experiments.

Several neuromodulators interact with STDP in parallel (Seol et al., 2007). Rat visual cortex layer 2/3 neurons exhibit no STDP for pairings in baseline conditions, but upon application of an agonist to noradrenaline receptors of the β family, a triangular STDP window manifests itself with LTP for both pre-before-post and post-before-pre pairings of up to 50 ms time difference (Seol et al., 2007). Conversely, the application of a M1-muscarinic acetylcholine agonist leads to LTD for both pre-before-post and post-before-pre. With a combination of agonists for both receptor types, the familiar STDP window is observed, where pairings pre-before-post lead to LTP, and reverse temporal order to LTD (see Figure 3D). A protocol where acetylcholine agonists are applied prior to pairing induces LTD, even if the agonists have been washed out 30 min before the start of the pairing; similarly, a protocol with noradrenaline agonists applied and washed out 40–50 min prior to pairing successfully induces LTP (Seol et al., 2007). The effect of the presence of neuromodulators *after* the pairing is not known.

In rat hippocampus pyramidal neurons, activation of noradrenaline receptors of the β family relaxes the timing constraint of pre-before-post pairings for the induction of LTP (Lin et al., 2003; see also Zhang et al., 2009).

In the drosophila mushroom body, the neuromodulator octopamine (thought to be functionally similar to noradrenaline) changes the STDP window from its “classic” shape to LTD for both timing orders (Cassenaer and Laurent, 2012).

In prefrontal cortex pyramidal neurons, acetylcholine receptor agonists (of the nicotinic receptor family) change normal pre-before-post pairings induced LTP to LTD (Couey et al., 2007). The apparent conflict with the results by Buchanan et al. (2010) and Sugisaki et al. (2011), who both find that activation of acetylcholine receptors facilitates timing-dependent LTP in rodent hippocampus, could arise from differences in brain region or other factors.

None of the above studies focuses on the precise timing of phasic neuromodulator signals. In hippocampus, the precise timing of cholinergic input plays an important role for LTP of synapses from Schaffer collaterals onto CA1 pyramidal neurons, when these synapses are driven by low-frequency pulses that normally are not efficient to induce plasticity (Gu and Yakel, 2011). However, how activity of the postsynaptic neuron would influence the picture was not tested.

In summary, experimental observations of neuromodulated STDP suggest a complex interplay of spike- and neuromodulator timing, concentrations and possibly other factors. Different neuron and synapse types in different brain regions may use different mechanisms. The experimental evidence with respect to the three questions raised above remains incomplete.

### 3.3. Traditional plasticity protocols in conjunction with neuromodulators

In plasticity experiments involving formation and remodeling of maps in sensory cortices, the exact timing of action potentials is not controlled. Instead the firing rate of neurons is indirectly manipulated by suitable stimulation and lesion paradigms. The influence of acetylcholine, noradrenaline, and serotonin on synaptic plasticity in sensory cortices with these classical rate-based paradigms has been reviewed before (Gu, 2002).

Traditional studies of dopamine-modulated plasticity in cortico-striatal synapses have also relied on “rate-based” protocols, where no particular attention is paid to the relative timing of pre- and postsynaptic spikes (see Reynolds and Wickens, 2002 and Jay, 2003, for reviews). For example, subthreshold intracellular current injection into striatal neurons together with simultaneous extracellular high-frequency stimulation of cortico-striatal fibers leads, under normal *in vitro* conditions, to LTD, but not when dopaminergic receptors of the D1/D5 or D2 family are pharmacologically blocked (Calabresi et al., 1992). In the absence of extracellular Mg^2+^, LTP is observed at normal dopamine levels (instead of LTD), but LTP induction is not possible if D1/D5 dopamine receptors are blocked (Kerr and Wickens, 2001). A summary picture in the way dopamine level modulates plasticity induction has been suggested by Reynolds and Wickens (2002), with high levels causing LTP, low levels LTD, and intermediate, as well as total absence of dopamine causing no changes at all (see Figure 3B).

## 4. Theories of modulated STDP

The scarcity of experimental data along with the complexity of the observed interactions of neuromodulators with synaptic plasticity pose a challenge to theoreticians: it is impossible at this stage to build and constrain plasticity models with the existing data. Moreover, while phasic neuromodulator signals, arising from e.g., dopaminergic or cholinergic neurons, are available in many brain regions, they act differentially on different neuron and synapse types (Gu, 2002). Given the variety of phenomena and the diversity of synapse types, a single unified model with one set of parameters is not to be expected. Instead, theoretical neuroscientists aim for a mathematical *framework* that would enable them to realize different plasticity phenomena by different choices of parameters in the same modeling framework.

As a first step toward such a framework, theoreticians ask fundamental questions such as: How *should* an individual synapse behave to become behaviorally relevant? What are *ideal* generalizations of the Hebbian learning principle, so that the brain as a whole would be able to solve complex learning tasks reinforced by reward, punishment, novelty, attention, or surprise?

Before we review theoretical approaches undertaken to answer these questions, we need to introduce the mathematical framework that will allow us a categorization of existing models of STDP under the influence of neuromodulation.

### 4.1. Formalization of modulated hebbian plasticity

While Hebbian learning rules have two main factors, i.e., the presynaptic activity and the state of the postsynaptic neuron, a synaptic plasticity rule that is influenced in addition by a neuromodulator will be called a “three-factor rule” in the following. In general, any three-factor synaptic plasticity rule incorporating neuromodulation, as well as pre- and postsynaptic activity, can be written as

(2) $$\begin{array}{l} \dot{w}=F\left(M,\text{pre},\text{post}\right) \end{array}$$

where $\dot{w}$ represents the weight change rate of a particular synapse from a pre- to a postsynaptic neuron. The variable *M* on the right-hand side is the modulator signal. Because it is typically received and shared by many synapses, its effect is sometimes called “heterosynaptic modulation” (Bailey et al., 2000). The variable *M* represents an *extrinsic* signal in the sense that it is generated neither by the synapse itself nor by the pre- and post-synaptic neurons (Marder, 2012). In the theoretical literature, the variable *M* is sometimes called a *global* factor in the sense that information conveyed by the time course of *M* is available to many (but not necessarily all) neurons and synapses in parallel (Izhikevich, 2007; Frémaux et al., 2013). As before, the acronyms “pre” and “post” represent the spike train of the pre- and the state of the postsynaptic neuron, respectively. In the theoretical literature, the variables summarized by “pre” and “post” are called the *local* factors of the synaptic update rule in the sense that the information conveyed by the spikes of one specific presynaptic neuron and the state of one postsynaptic neuron are available at the synapse (or synapses) connecting those two neurons (but not at other synapses). *F* is a function, the specifics of which determine the exact type of the learning rule. Since three-factor rules are a modern generalization of the original concept of Hebb, they are also called “neo-Hebbian” (Lisman et al., 2011).

Experiments that control presynaptic spiking, postsynaptic activity, and neuromodulation (see previous Section) roughly sketch the space of possible candidate functions that we could use for *F*. Since, however, data is scarce, no specific function *F* can be extracted at present from the experimental data. Instead, theoreticians have proposed potential candidate functions that could play the role of *F*. In particular, the function *F* of the three variables is sometimes assumed to consist of a “Hebb-like” term *F*_1_(*pre, post*) multiplied by a modulator function *g*_1_(*M*), hence $\dot{w}$ = *F*(*M*, pre, post) = *g*_1_(*M*)·*F*_1_(*pre, post*). Alternatively, the neuromodulator could directly change the postsynaptic activity, hence $\dot{w}$ = *F*(*M*, pre, post) = *F*_2_(*pre, post*(*M*)), but there are also other options.

In principle, the above mathematical framework of modulated synaptic plasticity should be applicable to various neuromodulators. For example, the phasic signal of noradrenaline-emitting neurons in locus coeruleus which has been linked to focused attentiveness on task-specific targets (Aston-Jones and Cohen, 2005) could influence synaptic plasticity and play the role of the modulator *M* in Equation (2). Similarly, in conditioning tasks, reward-related dopamine signals (Schultz et al., 1997; Schultz, 2002) can play the role of the modulator *M* in Equation (2). In particular, several recent studies have proposed models to link reward-based behavioral theories on one side, and models of learning at the level of individual neurons and synapses on the other side. In the following we focus on reward-driven learning models and cast them in the framework of the above three-factor rule.

### 4.2. Policy gradient models: R-max

One of several mathematical schemes to arrive at candidates for the function *F*, is to focus on the problem of reward-driven learning and derive a synaptic plasticity rule from the principle of iterative reward-maximization (Xie and Seung, 2004; Pfister et al., 2006; Baras and Meir, 2007; Florian, 2007; Di Castro et al., 2009; Urbanczik and Senn, 2009; Vasilaki et al., 2009; Frémaux et al., 2010). In the following, rules derived from reward maximization are called R-max. More specifically, R-max plasticity rules result from the application of policy gradient methods (Williams, 1992; Baxter and Bartlett, 2001) to a stochastically spiking neuron model. Synaptic “eligibility traces” arise from theoretical considerations and effectively bridge the temporal gap between the neural activity and the reward signal.

**Figure 4:**
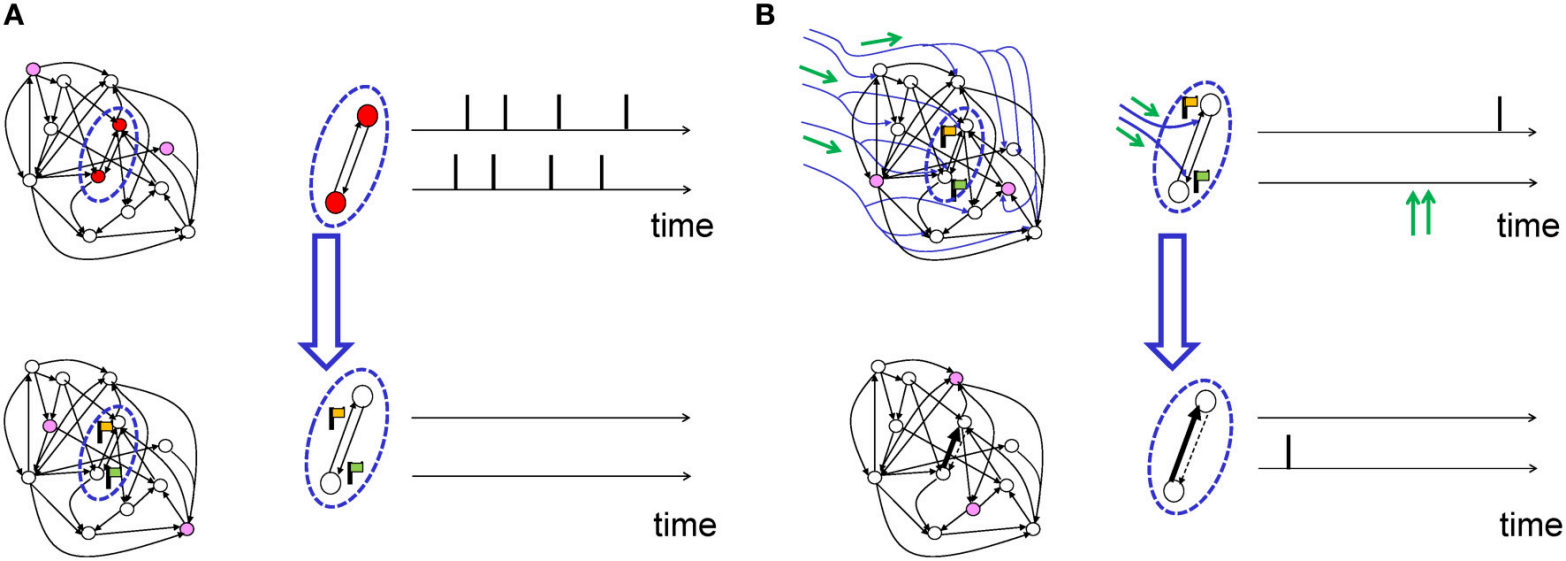
**Schematic of eligibility traces**. **(A)** Joint activity (top) of two neurons (red-filled circles mark active neurons, open circles inactive neurons) leads, a few moments later (bottom), to the raising of a “flag” at synapses connecting those two neurons. These synapses are now eligible for change. The activity of other neurons (filled pink circles) does not interfere with the flag, which persists over a short time. **(B)** Axonal branches of dopaminergic neurons are shown in blue. If a neuromodulatory signal (green arrows, top) arrives, the synapses with raised flags undergo plasticity (top: synapses of medium strength before phasic neuromodulatory signal; bottom: synapses after change). Bottom: In a model where the amplitude of STDP is amplified by the neuromodulator, a synapse becomes stronger (bold arrow) in case of earlier pre-before-post spike timing and weaker (dashed arrow) in case of reversed timing. Synapses remain stable thereafter.

Suppose a presynaptic neuron sends a spike train “pre” to a postsynaptic neuron with spike train “post.” Similar to Hebbian learning the synapse will form a transient memory of coincidences between pre- and postsynaptic spikes. This transient memory, called the “eligibility trace” in the theoretical literature and “tag” in the experimental literature, decays over a time scale τ_*e*_. While the transient memory persists, the synapse is marked and therefore eligible for changes later on (Figure 4A). The actual change of the synapse, however, requires in addition a neuromodulatory signal *M* (Crow, 1968). Conceptually, the neuromodulator could target a specific subset of synapses, or a large, but random fraction of synapses in the brain. We emphasize, that even if the anatomical branching patterns are unspecific, only the synapses which have been previously marked by the eligibility trace will be changed (Figure 4B).

In the theory of R-max, the eligibility trace evolves according to

(3) $$\begin{array}{l} \dot{e}=-\frac{e}{\tau_{e}}+\left(H\left(\text{pre},\text{post}\right)-\langle H\left(\text{pre},\text{post}\right)|\text{pre}\rangle \right), \end{array}$$

where 〈·|·〉 represents the conditional expected value and *H* is a Hebbian function, i.e., it denotes the joint activity of pre- and postsynaptic neurons. In the special case of a stochastically spiking postsynaptic neuron driven by excitatory postsynaptic potentials (EPSPs) arriving from one or several presynaptic neurons, the function *H* represents the value of the EPSP triggered by a presynaptic spike evaluated at the time of a postsynaptic spike: this is similar to the “pre-before-post” part of the STDP window (Figure 3A). The maximization principle used for the derivation of the R-max rule therefore makes a prediction for “pre-before-post” timing, but no prediction for “post-before-pre” (in fact, “post-before-pre” does not matter). While the specific prediction that the shape of the EPSPs should match the “pre-before-post” part of the STDP window is specific to one particular spiking neuron model, the principles of R-max could be generalized to other neuron models.

As mentioned before, the eligibility trace *e* marks the synapse for a change later on (Crow, 1968), but does not lead by itself to a change of the synaptic weight. The weight change requires the presence of a neuromodulator *M* and is proportional to

(4) $$\begin{array}{l} \dot{w}=M\times e \end{array}$$

where *M* is the neuromodulator. In R-max *M* could be equal to the reward (that is, *M* = *R*) or to the reward corrected by a bias *b* (that is, *M* = *R* − *b*).

**Figure 5:**
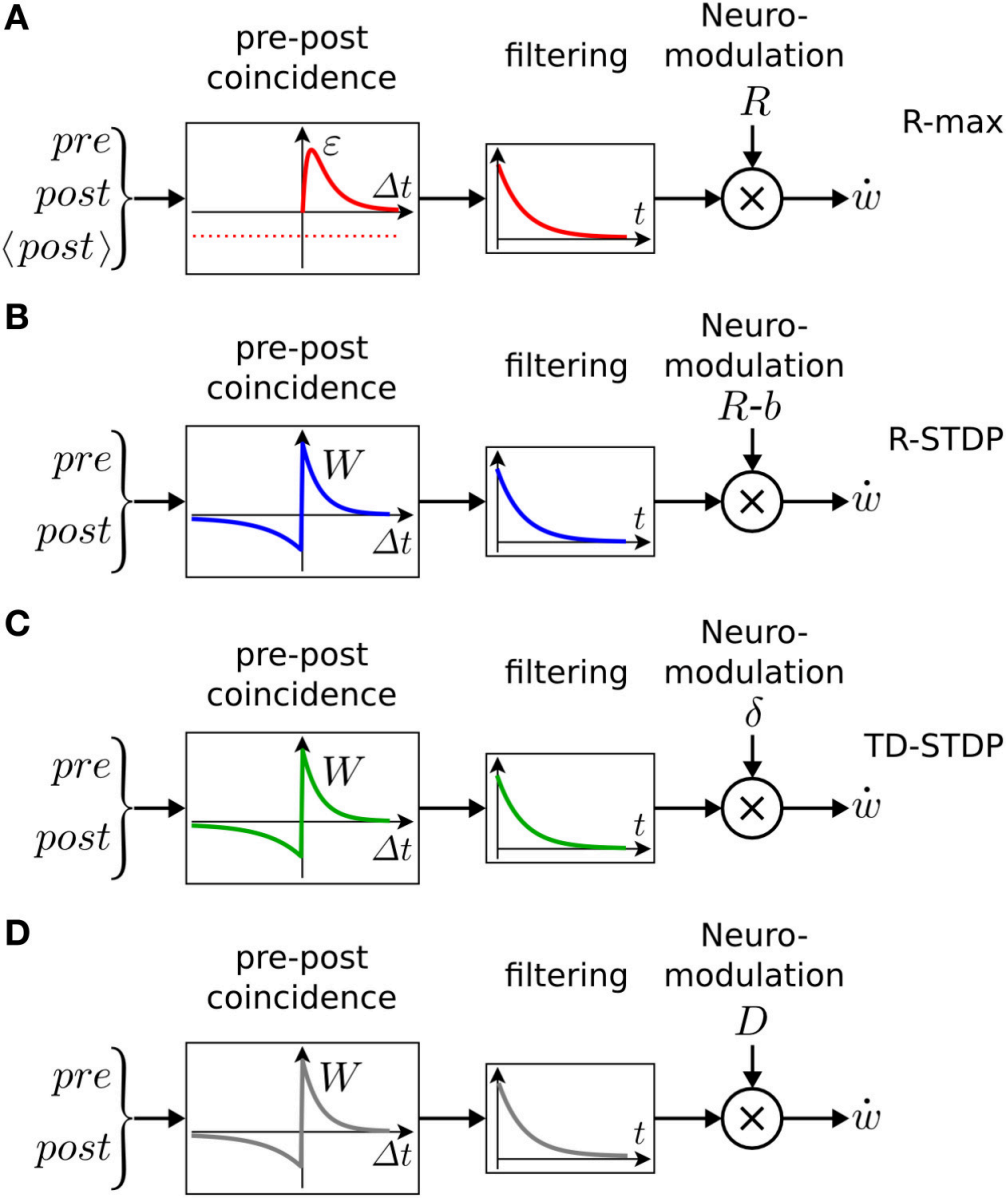
**Schematic of reward-modulated learning rules**. Boxes on the left show the magnitude of plasticity as a function of the time difference Δ*t* = *t*_post_−*t*_pre_, between post- and presynaptic spike firing. **(A)** R-max (Pfister et al., 2006; Baras and Meir, 2007; Florian, 2007; Frémaux et al., 2010). The learning rule is maximal for “pre-before-post” coincidences (red line, ϵ) and rides on a negative bias representing the expected number of postsynaptic spikes 〈*post*〉 (red dashed line). This Hebbian coincidence term is then low-pass filtered by an exponential filter, before being multiplied by the delayed reward *R* transmitted by a neuromodulator. **(B)** R-STDP (Farries and Fairhall, 2007; Florian, 2007; Izhikevich, 2007; Legenstein et al., 2008; Vasilaki et al., 2009; Frémaux et al., 2010). Similar to A, except for the shape of the pre-post coincidence window *W* which is bi-phasic and does not depend on the expected number of postsynaptic spikes. The Hebbian coincidence term is, after filtering, multiplied with the neuromodulator transmitting the success signal *M* = *R* − *b* where *b* is the expected reward. **(C)** TD-STDP (Frémaux et al., 2013). Similar to B, except for the modulating factor which in this case is the TD error *M* = δ^TD^. **(D)** Generalized learning rule. Changing the meaning of the neuromodulator term *M* = *D* allows the switching between different regimes of the learning rule.

The learning rule of Equations (3) and (4) can be qualitatively described as follows (see also Figure 5A). The term *H*(pre, post) detects the coincidence between a pre-synaptic and a post-synaptic spike, where the timing requirement for the coincidence is controlled by the duration ϵ of the EPSP. The expected number of coincidences, 〈*H*(pre, post)〉, is subtracted: the result (symbolized by the left box in Figure 5A) is hence the deviation of the observed pre-post coincidences from the expected number of pre-post coincidences. This deviation is then low-pass filtered with an exponential-shaped function to yield the eligibility trace *e*, before being multiplied by reward. The time constant τ_*e*_ of the exponential arises from the integration of Equation (3) and determines the maximal interval between the pre-post coincidences and the reward. A large value of τ_*e*_ implies that coincident activity of pre- and postsynaptic neurons that occurred a long time back in the past is still memorized by the synapses at the moment when a reward is received. An eligibility trace with a long time constant τ_*e*_ allows therefore to bridge the temporal gap between Hebbian coincidences (that occurred at the moment when the action was chosen) and reward delivery (Figure 4). A small value of τ_*e*_, however, implies that the reward must be concurrent with, or at most be slightly delayed compared to neural activity.

R-max is an example of a covariance rule (Loewenstein and Seung, 2006) because the expected evolution of synapses is sensitive to the covariance between the reward *R* and the Hebbian term

(5) $$\begin{array}{l} \langle \dot{w}\rangle =\mathrm{Cov}\left(R,H\left(\text{pre},\text{post}\right)\right) \end{array}$$

where the covariance is analyzed on the time scale τ_*e*_. Covariance rules have generic properties that have been related to the matching law of operant conditioning (Loewenstein and Seung, 2006; Loewenstein, 2008).

### 4.3. Phenomenological models: R-STDP

While the learning rule discussed in the previous section can be rigorously derived from optimization principles (“policy gradient”), other rules based on more heuristic considerations have been proposed. A prominent example is reward-modulated STDP (R-STDP) which has been studied in several publications (Farries and Fairhall, 2007; Florian, 2007; Izhikevich, 2007; Legenstein et al., 2008; Vasilaki et al., 2009; Frémaux et al., 2010; Friedrich et al., 2011).

The main idea is to modulate the outcome of “standard” STDP (left box in Figure 5B) by a reward term. A synaptic eligibility trace (middle box in Figure 5B) stores a temporary memory of the STDP outcome so that it is still available by the time a delayed reward signal is received. If one writes the timing condition (or “learning window”) (Gerstner et al., 1996; Kempter et al., 1999; Abbott and Nelson, 2000; Song et al., 2000) of traditional Hebbian STDP as STDP(pre, post), the synaptic eligibility trace keeps a transient memory in the form of a running average of recent spike-timing coincidences

(6) $$\begin{array}{l} \dot{e}\;=\;-\;\frac{e}{\tau_{e}}\;+\;\text{STDP}\left(\text{pre},\text{post}\right) \end{array}$$

where τ_*e*_ is the time constant of the eligibility trace. The running average is equivalent to a low-pass filter (middle box in Figure 5B).

In R-STDP, the synaptic weight *w* changes when the neuromodulator *M* = *R* − *b* signals a deviation of the reward *R* from a baseline *b*,

(7) $$\begin{array}{l} \dot{w}=\left(R-b\right)\times e. \end{array}$$

In most publications, the baseline is chosen equal to the mean reward *b* = 〈*R*〉 which makes R-STDP a covariance rule. Indeed, if the baseline *b* in Equation (7) is different from the mean reward, the learning rule does not function properly as a reward-based learning rule (Frémaux et al., 2010).

If we integrate Equation (6), we can write *e* as the running average over past spike-time coincidences $e=\bar{\text{STDP}}\left(\text{pre},\text{post}\right)=H\left(\text{pre},\text{post}\right)$. In this case, R-STDP can be summarized in a single equation

(8) $$\begin{array}{l} \dot{w}=\left(R-\langle R|\text{pre}\rangle \right)\times H\left(\text{pre},\text{post}\right) \end{array}$$

where the baseline is the expected reward and the Hebbian term *H*(pre, post) is the running average of past spike-timing coincidences. Our notation of the mean reward 〈*R*|pre〉 emphasizes that the mean reward must be evaluated in a stimulus-specific fashion.

How could the brain evaluate a mean reward? In the simplest case, the mean reward could be a running average over past experiences. Indeed, if an agent repeats the same learning task many times, the running average of past rewards is an excellent approximation of the mean reward and the agent can learn a complex behavioral task (Frémaux et al., 2010). However, a simple running average is useless if the agent has to learn two (or more) tasks in parallel: in a nontrivial situation, different learning tasks have different rewards, but the running average would smooth out these differences, so that none of the tasks is learned (Frémaux et al., 2010; Herzog et al., 2012).

To achieve learning of multiple tasks in parallel, the running average has to be task-specific ${\overset{ˉ}{R}}_{\text{task}}=\langle R|\text{task}\rangle$ (Frémaux et al., 2010). If R-STDP is implemented with task-specific averaging of the reward, R-STDP turns into an example of a covariance rule

(9) $$\begin{array}{l} \langle \dot{w}\rangle =\mathrm{Cov}\left(R,H\left(\text{pre},\text{post}\right)\right). \end{array}$$

This is equivalent to Equation (5), i.e., R-max and R-STDP with mean reward subtraction both compute the covariance of reward and a Hebbian term *H*.

R-STDP with a neuromodulatory signal $M=R-{\overset{ˉ}{R}}_{\text{task}}$ is the most widely used form of reward modulated STDP (Farries and Fairhall, 2007; Florian, 2007; Legenstein et al., 2008; Vasilaki et al., 2009; Frémaux et al., 2010; Friedrich et al., 2011). However, Izhikevich has studied a different scenario which we call “gated-Hebbian” learning: sparse, positive rewards are given to reinforce the occurrence of pre-before-post spiking events at particular synapses (Izhikevich, 2007). In that case Equation (9) does not hold, but instead we have

(10) $$\begin{array}{l} \langle \dot{w}\rangle =\mathrm{Cov}\left(R,H\left(\text{pre},\text{post}\right)\right)+\langle R\rangle \;\langle H\left(\text{pre},\text{post}\right)\rangle . \end{array}$$

Izhikevich balances the STDP window and the network activity so that 〈*H*(pre, post)〉 is slightly negative: combined with positive rewards (〈*R*〉 > 0), the second term on the left-hand side of Equation (10) is negative and causes a downward drift of all synaptic weights.

If rewards are given conditionally on the occurrence of a specific pre-before-post spike pairing “target,” the covariance term in the left-hand side of Equation (10) is zero for all connections, except for the one single synapse that represents the target pair. The above form of R-STDP is therefore successful in a task, where the goal is to strengthen a particular target synapse and depress all the others. In other words, in the R-STDP model of Equation (10), rewards are used as a binary *gating* signal to switch from general synaptic depression to the potentiation of a particular synapse.

In summary, R-STDP relies on two critical assumptions. First, Hebbian plasticity is modulated by reward. Evidence for the relation of reward to dopamine and the modulation of STDP by dopamine has been reviewed above. Second, synapses are marked by eligibility traces to enable the bridging of the temporal gap between Hebbian coincidences and the occurrence of the reward signal. An eligibility trace is a transient memory of past Hebbian coincidence events stored at the location of the synapse. The biological plausibility of eligibility traces and its potential relation to synaptic “tags” (Frey and Morris, 1997; Bailey et al., 2000; Redondo and Morris, 2011) will be explored in the discussion section.

### 4.4. Temporal-difference learning with STDP

There is a strong conceptual similarity between the temporal difference (TD) error which arises in reinforcement learning and the patterns of activity of dopaminergic neurons during experiments involving rewards (Schultz et al., 1997; Waelti et al., 2001; Schultz, 2002; Doya, 2007). In TD learning methods (Sutton and Barto, 1998), the environment in which the animal evolves is described in terms of states. The transitions from one state to the next are determined by the choice of an action. To each state, a value is assigned (state value), which corresponds to the amount of future reward that can be expected if the animal starts from that particular state. Given the correct value for each state, the optimal policy consists in choosing the action that leads to the available state with the highest value. The key problem to solve is thus to learn the correct values associated with each state.

Naively, one would attempt to solve this problem by running many trials starting from a given state, and observing the average reward return obtained. However, TD methods (Sutton and Barto, 1998) solve this problem more efficiently using algorithmic ideas inspired by dynamic programming (Bellman, 1957; Bertsekas, 1987): Consistency of state values across different states requires that the expected reward in one state (visited at time *t*) be equal to the mean reward obtained during the transition to the next state *plus* the reward expected in the state visited at time *t*+1. This consistency relation should hold for the correct values: if the agent does not yet know the true values, but works with momentary estimates, the mismatch δ^TD^ of the consistency relation, called the temporal difference (TD) error, is

(11) $$\begin{array}{l} \delta^{\text{TD}}=“\text{value expected at }t+1”\text{ ​+ ​}“\text{reward at transition to }t\text{ + 1}”\\ \;-\text{ value expected at }t”. \end{array}$$

If the estimated state values are updated using the information contained in δ^TD^, the estimated state values will eventually converge to the true solution (Dayan, 1992). Updates proportional to the TD error are the essence of TD learning.

Early modeling studies linking TD and the brain do not use spiking neurons, but instead rely on networks of dynamic systems to explain how TD computation can be linked to anatomical structures (Houk et al., 1995; Suri and Schultz, 1998, 1999, 2001; Joel et al., 2002). Other studies focused on implementing reinforcement learning algorithms in artificial neural networks, in particular for navigation problems (Arleo and Gerstner, 2000; Foster et al., 2000; Sheynikhovich et al., 2009).

In implementations of simulated neural networks, the state values are often represented in a substructure called the “critic,” from which the TD error is extracted; the choice of actions takes place in a different substructure called the “actor.” Recently, Potjans et al. (2009, 2011) have used an actor-critic network of leaky integrate-and-fire neurons to solve a simple 5 × 5 grid-world task with spiking neurons. They propose novel, non-STDP learning rules which make explicit use of discrete state transitions.

In Frémaux et al. (2013), a TD learning rule for spiking neurons is derived analytically, which has the form

(12) $$\begin{array}{l} \dot{w}=\delta^{\text{TD}}\times H\left(\text{pre},\text{post}\right), \end{array}$$

where δ^TD^ is a continuous time version of the TD error, and *H* is the eligibility trace and accounts for a running average of Hebbian coincidences between the pre- and postsynaptic activities.

The analytically derived Hebbian term is a pre-before-post coincidence window with the shape of an EPSP. However, using a bi-phasic STDP window (left box in Figure 5C) leads to a valid, and well-functioning, learning rule, which we denote as TD-STDP.

### 4.5. Beyond rewards: other models of three-factor learning rules

In all of the above examples, we have focused on models of reward-based learning with dopamine as the candidate neuromodulator (Schultz et al., 1997; Waelti et al., 2001; Steinberg et al., 2013). The general framework of three-factor rules (Equation 2) can, however, also be applied to a variety of learning paradigms where the role of the neuromodulator *M* could be different. For example, for the learning of binary decisions in populations of spiking neurons, a neuromodulatory signal proportional to the population activity has been suggested (Urbanczik and Senn, 2009; Friedrich et al., 2011). The neuromodulator encodes the population decision and allows individual neurons to compare their private spiking history with the decision of the population. While such a scheme can help in binary decision making and is biologically plausible, it is not clear how it can generalize to non-binary decision making problems, such as motor learning. Another example is the learning of complex sequences in spiking neural networks with several layers. Learning is most efficient if it is triggered by a “surprise” signal which conveys novelty of the observed state compared to expected novelty (Brea et al., 2013; Rezende and Gerstner, 2014; see also Schmidhuber, 1991). For example in Rezende and Gerstner (2014), the weight changes directly depend on a Hebbian function *H* multiplied with a neuromodulator *S* that conveys surprise, i.e., $\dot{w}$ = *S*·*H*(*pre, post*), Phasic signals of neuromodulators that reach a large fraction of neurons in the brain are good candidates for transmitting such a surprise or curiosity signal that gates plasticity (Gu, 2002; Lisman et al., 2011; Gruber et al., 2014). At the present stage of molecular knowledge, detailed models of molecular mechanisms can be at most of a hypothetical nature (Nakano et al., 2010).

## 5. Discussion

### 5.1. A general framework for reward-modulated STDP

The learning rules reviewed above (Equations 3, 4, 8, and 12) broadly fall in two different classes. The first class contains covariance-based learning rules, such as R-max (Equation 3) or R-STDP as in Equation (8). These learning rules move the synaptic weights *in the mean* over many trials. In any single trial, stochasticity of the postsynaptic neuron is needed to make the agent explore different actions. The covariance between neural activity and reward will eventually drive learning in the right direction, via a running average over a large number of trials. For that reason covariance-based rules are slow: they typically need thousands of trials to converge to a good solution.

The second class consists of spike-timing dependent variants of TD-learning such as TD-STDP (Equation 12), as well as the gated scenario of R-STDP (Equation 10). For learning rules in this class, weight updates after a single trial typically move the synaptic weights in the desired direction. This implies that learning is possible after just a few trials. However, in the case of TD learning, the presynaptic neurons must provide a useful representation of the state of the agent. How such representation can be learned (using some variant of unsupervised learning) is not part of standard reinforcement learning theory.

From the point of view of synaptic plasticity, all of the above learning rules can be implemented as a three-factor rule

(13) $$\begin{array}{l} \dot{w}=M\times H\left(\text{pre},\text{post}\right), \end{array}$$

where *M* represents the neuromodulator (third factor), and *H* is the running average of Hebbian coincidences, measured either through the standard bi-phasic STDP window, or the pre-before-post coincidence window only. To switch between the different variants of neuromodulated spike-timing dependent learning, the neuromodulator *M* has to take on different roles:

(14) $$\begin{array}{l} M=\left\{ \begin{array}{l} R-\langle R\rangle \;\to \text{ covariance-rule }\\ \delta^{\text{TD}}\;\to \text{ TD learning }\\ R\;\to \text{ gated Hebbian learning}\\ S\;\to \text{ surprise/novelty-modulated STDP},\\ const\;\to \text{ non-modulated STDP}, \end{array}\right. \end{array}$$

where *S* is a measure for surprise, novelty, or curiosity and *const* denotes some positive constant. While the first three cases fall in the class of reward-based learning, the fourth one represents curiosity or surprise driven learning. The last case (with constant factor) represents standard unsupervised STDP (or other voltage or rate-dependent variants of Hebbian learning) where the action of neuromodulators is irrelevant. The similarity of different three-factor rules (Figure 5D) raises the possibility that, depending on brain region and neuron type as well as on the availability of various neurotransmitter, slight modifications of the same molecular plasticity mechanisms could implement different learning schemes.

### 5.2. Subtraction of the expected reward

There are similarities, but also subtle differences between the five different roles that the neuromodulator *M* takes in Equations (13) and (14). In the first and second line of Equation (14) the neuromodulatory term *M* can be described as “actual reward minus expected reward,” similar to the formulation of the activity of dopaminergic neurons (Schultz et al., 1997; Schultz, 2002). However, in the first line the term “expected” takes a different meaning from that in the second line. In the covariance form of R-STDP (first line in Equation 14), “expected” refers to the statistical expectation of the reward. In practice, the expected reward can be implemented as a (task-dependent) running average over previous trials, as discussed earlier. In the case of TD-STDP (second line in Equation 14), however, “expected” is to be understood in the sense of *predicted*. In practice, reward prediction requires that for each state (or each state-action pair) expected reward values are estimated.

These differences have important consequences for learning. The covariance form of R-STDP can function properly only if the neuromodulatory signal *M* is zero on average. This requires the expected reward 〈*R*〉 to be known. If a running average of the reward is used as an approximation to the statistical expectation, the running average must converge *before* correct learning can occur. In contrast, TD-STDP will only learn while the neuromodulatory signal (the TD error) is *not* equal to zero. As soon as the state values have converged to their correct values, the TD-error vanishes and so does the neuromodulatory signal. Learning thus only occurs *during* convergence of the value estimation; after convergence, learning stops. The fact that convergence in TD and covariance learning occurs on different timescales (fast for TD, slow for covariance, see Frémaux et al., 2013) suggests that the two might be used in a combined manner. The feasibility of such a learning system deserves further study.

Experimental data on phasic dopamine signals are consistent with the notion of “actual reward minus expected reward” once the dopamine baseline is subtracted (Schultz et al., 1997). Indeed, blocking experiments show that learning of compound stimuli reappears when a phasic dopamine signal is artificially switched on (Steinberg et al., 2013), but remains blocked in the absence of phasic dopamine (Waelti et al., 2001). The fact that dopaminergic neurons also fire in response to reward-predicting stimuli (which are not rewarding by itself!) suggests that dopaminergic firing contains information related to a TD error (Schultz et al., 1997). Recently, mechanistic ideas of how such a TD signal could be calculated in the brain have started to appear (Cohen et al., 2012). Interestingly, phasic and bi-phasic responses of dopamine to novel stimuli suggest that dopamine may also transmit novelty related information (Schultz, 1998; Waelti et al., 2001; Redgrave and Gurney, 2006; Lisman et al., 2011).

### 5.3. Eligibility traces and synaptic tagging

An eligibility trace is a transient memory of past Hebbian coincidence events stored at the location of the synapse. Eligibility traces are an essential part of most three-factor learning rules, Equation (13), because they bridge the temporal delay between the sensory input and/or action on the one side and the moment of reward delivery on the other side.

From a theoretical point of view, eligibility traces appear for a variety of reasons. One of them is the extension of finite horizon policy gradient methods to so-called infinite horizon problems (Baxter and Bartlett, 2001; Pfister et al., 2006). In that case, a decaying trace is used to set the time horizon, with the heuristics that recent policy choices should get more credit for rewards than distant ones. A similar argument, albeit with a weaker theoretical foundation, is made in the case of reward-modulated STDP with eligibility traces (Klopf, 1982; Seung, 2003; Farries and Fairhall, 2007; Florian, 2007; Izhikevich, 2007): synapses that underwent pairing just before a reward should get a stronger reinforcement than those that underwent pairing earlier. In standard TD-learning, eligibility traces are sometimes added “*ad-hoc*” to speed up learning (Sutton and Barto, 1998). In spiking networks, eligibility traces arise directly from the need of extracting a smooth signal from spike trains, in order to be able to derive a TD error minimizing learning rule (Frémaux et al., 2013).

From a functional perspective, eligibility traces fulfill a similar role as the synaptic tagging mechanism of Frey and Morris (1997, 1998). In experiments on synaptic tagging and capture (Frey and Morris, 1997, 1998; Redondo and Morris, 2011), *strong* stimulation of hippocampal neurons through a presynaptic input is sufficient to elicit late LTP, whereas *weak* stimulation only causes a transient synaptic efficacy change (early LTP) that decays on the time scale of 1 or 2 h. However, when the neuron is subject to both strong and weak stimulation at two different presynaptic pathways, both sets of synapses get consolidated (Frey and Morris, 1997). Crucially, this happens even if the weak stimulation happens 1 h prior to the strong stimulation, suggesting the weakly stimulated synapses keeps a slowly decaying “tag” (Frey and Morris, 1998). Models of tagging and consolidation (Clopath et al., 2008; Barrett et al., 2009) further highlight the structural similarities between eligibility traces in reward-based learning and synaptic tagging. However, there are notable differences between synaptic tagging and eligibility traces, most prominently the different time scales of the synaptic memory traces: for tagging the decay of traces occurs on the time scale of ~1 hr (Frey and Morris, 1997; Reymann and Frey, 2007; Redondo and Morris, 2011), whereas it is in the range of a few hundred milliseconds for eligibility traces in reinforcement learning (Arleo and Gerstner, 2000; Foster et al., 2000; Izhikevich, 2007; Sheynikhovich et al., 2009; Frémaux et al., 2013). Nevertheless, the fact that the molecular machinery necessary to maintain synaptic traces exists (Lisman et al., 2011) exists in the context of synaptic consolidation, also lends biological plausibility to the concept of eligibility traces: the implementation of eligibility traces could use a signaling chain that is analogous to that for synaptic consolidation, but with shorter time constants. The timing requirement between spike pairings and dopamine deserves additional experimental investigation.

### 5.4. Role of the post-before-pre part of the STDP window

All theoretical studies of STDP from an “optimality perspective” highlight the relevance of the pre-before-post part of the STDP window, but do not reliably predict a significant post-before-pre effect (Pfister et al., 2006; Bohte and Mozer, 2007; Toyoizumi et al., 2007; Parra et al., 2009). The fundamental reason is the direction of causation: pre-synaptic spikes only affect *later* post-synaptic spikes and (classic unsupervised) STDP detects these causal relations and makes them even stronger. The same argument of causality also applies to reward-modulated forms of STDP (Pfister et al., 2006; Florian, 2007). In practice, it was found that including a post-before-pre part of the learning window generally does neither help nor harm (Izhikevich, 2007; Farries and Fairhall, 2007; Frémaux et al., 2010, 2013). The main reason to include a post-before-pre dependency is that for unsupervised (i.e. not modulated) STDP, a bi-phasic learning window was found (e.g., Markram et al., 1997; Bi and Poo, 1998). However, for the case of dopamine modulated STDP, the currently available experimental evidence for the role of post-before-pre pairings is inconclusive (Pawlak et al., 2010). Thus, the role of the post-before-pre part of reward-modulated learning rules remains an open question for further theoretical and experimental studies.

### 5.5. Implications for the search of experimental evidence

Experimental evidence for the interaction of STDP with neuromodulation is still incomplete. This provides an opportunity for theoreticians to identify the most critical aspects that would be worth further experimental study.

The precise timing of a phasic neuromodulatory signal with respect to spike pairing is a crucial element in all theoretical models. While stimulation of dopaminergic axon bundles with bipolar electroces is a traditional means to elicit phasic dopamine release, it has now also become possible to to control neuromodulatory signals with optogenetic methods (see e.g., Tsai et al., 2009; Steinberg et al., 2013, for optogenetic activation of phasic dopamine signals in behaving animals). First steps have been taken in Gu and Yakel (2011), who control the precise timing of the neuromodulator and the presynaptic neuron, but not that of the postsynaptic cell. Instead of precise control of the neuromodulatory timing, a number of studies (e.g., Seol et al., 2007; Pawlak and Kerr, 2008; Zhang et al., 2009) focus on the precise relative timing of the pre- and postsynaptic spikes. There is no strong theoretical prediction as to the exact shape of either part of the STDP window, except that the time-dependence (or shape) of the pre-before-post window should roughly match the shape of an EPSP.

The reversal of plasticity under negative reinforcement is another critical feature of most theoretical models of reward-modulated STDP. This means that a spike pairing that would result in LTP under positive reinforcement would result in LTD under negative reinforcement and vice versa. Limited experimental evidence of this phenomenon exists (Reynolds and Wickens, 2002; Seol et al., 2007; Zhang et al., 2009) but more experiments are necessary to test the theoretical prediction that negative reinforcement should reverse the polarity of synaptic plasticity.

The existence of a “critic” structure in the brain is suggested in (i) theories of covariance-based learning, because an accurate, task-dependent reward prediction system is necessary (Frémaux et al., 2010), and by (ii) actor-critic architecture-based models of TD-learning, because the TD error needs to be calculated (Potjans et al., 2009, 2011; Frémaux et al., 2013). Strong evidence for such a critic structure is provided by the research by Schultz et al. (e.g., Ljunberg and Schultz, 1992; Schultz et al., 1993; Hollerman and Schultz, 1998; Waelti et al., 2001): since the dopamine signal represents “reward—expected reward,” some upstream structure must calculate the expected reward. Experimentally found activity of ventral striatum neurons in a maze navigation task (van der Meer and Redish, 2011) closely resembles that of simulated critic neurons (Frémaux et al., 2013). This further strengthens the case for the existence of a TD-based critic. Herzog et al. (2012) raise the possibility that the critic can be misled when two tasks are very similar, yet yield different mean rewards, which leads to the blocking of learning. This interesting possibility opens the way for psychophysics experiments probing the functioning of a potential critic structure in more detail.

**Figure 6:**
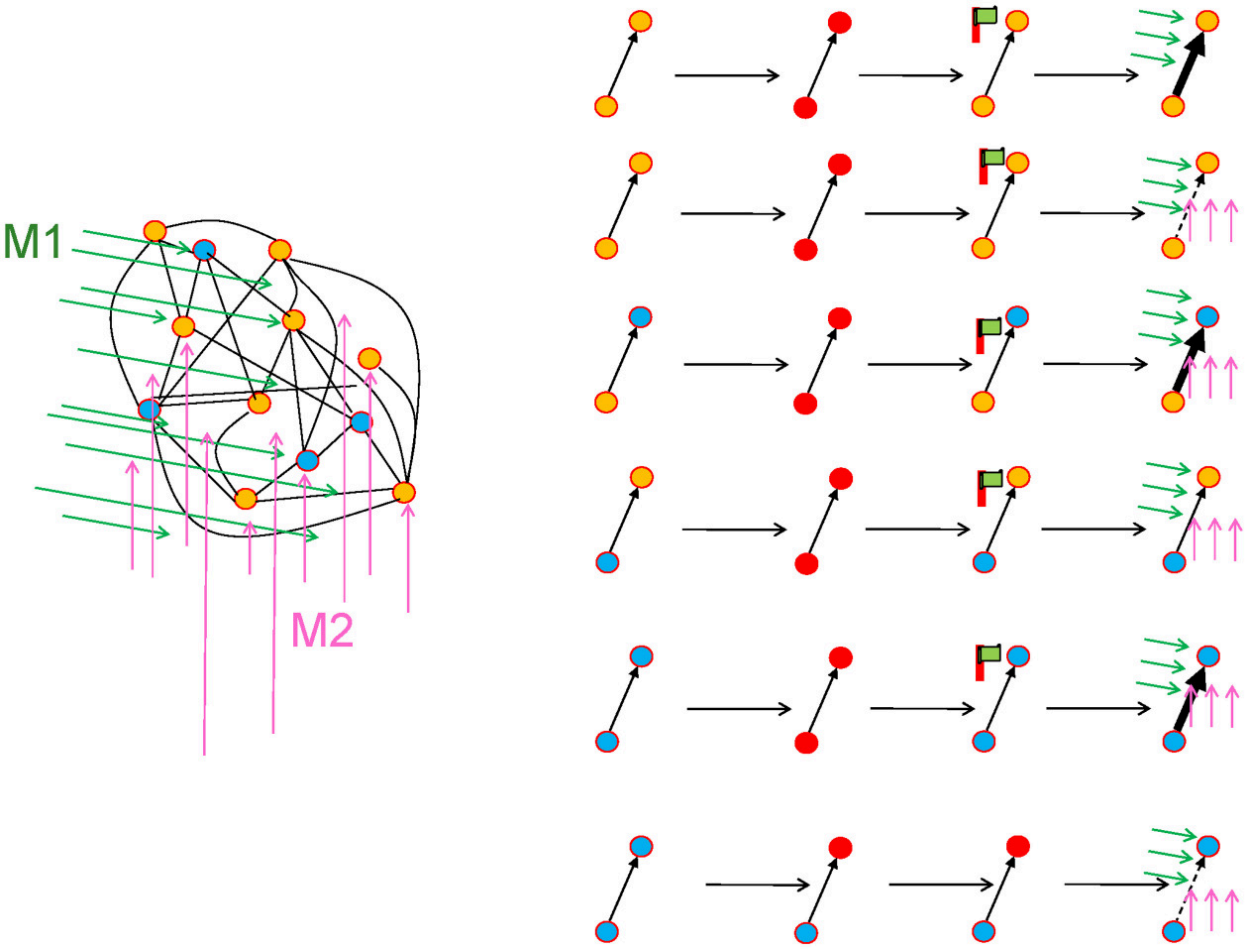
**Specificity of synaptic changes (schematic)**. In a network with several neuron types (here two types, orange and blue filled circles) and several neuromodulators (green arrows for neuromodulator M1 and magenta for M2), synaptic changes can be highly specific, even if phasic signals of neuromodulators have broad and unspecific target regions. Hypothetical examples, from top to bottom (red = active neuron): A connection from orange to orange becomes stronger (bold arrow), if both neurons and neuromodulator M1 are active; the same connection becomes weaker (dashed arrow), if in addition M2 is active; a connection from orange to blue is strengthened if both neurons and both neuromodulators are active; in the same situation, a connection from blue to orange is not affected; a connection from blue to blue is strengthened if both neurons and both neuromodulators are active; the same connection becomes weaker if only the postsynaptic neuron is active.

The role of neuromodulators is likely to be diverse. Even in the extreme case often considered by theoreticians where phasic neuromodulatory signals are available unspecifically to all synapses (which we do not believe to be true in the brain), neurons and synapses can be targeted specifically (Disney et al., 2006, 2007) given a set of plasticity rules that depend on (i) activity and type of presynaptic neuron; (ii) activity and type of postsynaptic neuron; (iii) the received mixture of neuromodulators (Figure 6). Dopamine is the neuromodulator most often associated with reward signals, but other important molecules include acetylcholine, noradrenaline and serotonin. These various neuromodulators might carry different signals separately or even together (Figure 1G) (Schultz, 1998; Waelti et al., 2001; Redgrave and Gurney, 2006; Lisman et al., 2011) and synapses may react to the specific mix of neuromodulators (Seol et al., 2007; Svensson et al., 2001; Katz and Edwards, 1999). Candidate roles for neuromodulatory signals predicted by theory include population decision signals (Urbanczik and Senn, 2009; Friedrich et al., 2011), reinforcement learning meta-parameters (Doya, 2002), or novelty-based “surprise” (Schmidhuber, 1991; Rezende and Gerstner, 2014). From a theoretical perspective, there is no need of a one-to-one mapping from neuromodulators to specific functions, but a mixed coding scheme would be sufficient (Figure 1G).

Models of behavioral learning associate abstract representations of sensory input to motor outputs (see e.g., Figure 1B). Reward-based learning with TD methods requires a *compact* representation of states (derived from sensory input) and actions (output), and becomes intractable when the number of states and actions become large (Sutton and Barto, 1998). In contrast, policy gradient methods do not need input and output complexity reduction in order to work properly, but they converge faster on a compact representation (Farries and Fairhall, 2007; Frémaux et al., 2010). Unsupervised learning methods (e.g., Kohonen, 1990; Hinton and Sejnowski, 1999; Franzius et al., 2007) are one way to achieve compact representations of inputs in complex learning tasks (Arleo and Gerstner, 2000; Arleo et al., 2004; Swinehart and Abbott, 2006; Franzius et al., 2007; Sheynikhovich et al., 2009). It remains an open question whether the development of compact representations of sensory input in the brain is purely unsupervised Hebbian (as it seems to be the case, e.g., in inferotemporal cortex, Li and DiCarlo, 2012), or whether, in some brain areas, it is also influenced by reward and novelty information.

#### 5.5.1. Outlook

Most of the model networks studied in this review consist of layers of homogeneous neurons connected to each other in a simple “feed-forward” structure. In contrast the brain features highly recurrent networks of various neuron types. Recurrent networks could, in principle, provide a rich reservoir of activity states (Buonomano and Maass, 2009; Sussillo and Abbott, 2012) and implement complex computations (Nessler et al., 2013; Sussillo, 2014). How learning of the *recurrent* connections (Laje and Buonomano, 2013; Hennequin et al., 2014) can be achieved by STDP under neuromodulation, while maintaining sustained balanced network activity (van Vreeswijk and Sompolinsky, 1996; Brunel, 2000; Vogels et al., 2005) remains an open question.

The multiple ways in which neuromodulators can interact with neural activity (Kaczmarek and Levitan, 1987; Nicola et al., 2000; Marder, 2012; Nadim and Bucher, 2014) combined with complex network structures suggest many different mechanisms of interplay between them (Marder, 2012). In this review we only focused a small subset of questions that relate to long-term synaptic plasticity. Even there, we have been coarse—for example we did not mention the additional complexity induced by the D1 and D2 dopaminergic receptor families, known to be expressed in different proportions across types of neurons (Missale et al., 1998; Seamans, 2007; Pawlak and Kerr, 2008; Shen et al., 2008). Dopamine receptors in turn are coupled to G-proteins linking to a large family of signaling cascades (Nicola et al., 2000).

We also neglected the direct influence of neuromodulators on synaptic strength and short-term plasticity (Nadim and Bucher, 2014). In many cases, theoretical models should simplify, and therefore neglect complexity, in as much as they aim to extract simple functional rules, or concepts, linking neuromodulation and plasticity. However, for the purpose of reproducing and understanding biophysical experiments and, e.g., predicting the manifold actions of pharmacological drugs and their interactions with neurotransmitters, detailed models of neuromodulated plasticity will eventually be needed.

### Conflict of interest statement

The authors declare that the research was conducted in the absence of any commercial or financial relationships that could be construed as a potential conflict of interest.
